# Treatment of Uveal Melanoma Using a Novel Radiosurgical Platform: A Case Report

**DOI:** 10.7759/cureus.81343

**Published:** 2025-03-28

**Authors:** Stephan Kinzl, Florian Heussen, Klemens Paul Kaiser, Ute Hornberger, Matthias D Becker, Andreas Mack, Dirk Weltz, Maya Müller, Boris Dettinger, Christina Picardi, Jürgen Curschmann

**Affiliations:** 1 Ophthalmology, Stadtspital Zürich, Zurich, CHE; 2 Ophthalmology, Inselspital, Bern University Hospital, Bern, CHE; 3 Ophthalmology, Goethe University, Frankfurt am Main, Frankfurt am Main, DEU; 4 Radiosurgery, Swiss Neuro Radiosurgery Center, Zurich, CHE; 5 Physics, Swiss Neuro Radiosurgery Center, Zurich, CHE; 6 Radiation Oncology, Swiss Neuro Radiosurgery Center, Zurich, CHE; 7 Radiation Oncology, Klinik Hirslanden, Zurich, CHE

**Keywords:** mechanical vacuum eye fixation, radiotherapy, stereotactic radiosurgery, uveal melanoma, zap-x® radiosurgery

## Abstract

We report on a patient with uveal melanoma treated with radiosurgery using the ZAP-X^®^ system (Zap Surgical Systems Inc., San Carlos, USA), a novel stereotactic radiosurgery delivery system dedicated to the treatment of intracranial lesions.

A 78-year-old female patient presented with a collar-button uveal melanoma measuring 9.90 mm in the largest basal diameter and 12.97 mm in ultrasonographic thickness with associated neurosensory detachment. The gross tumor volume (GTV) in the right eye was 1.47 cm^3 ^and was defined geometrically and stereotactically in three dimensions using imaging data. The planning target volume (PTV) was created by adding a 2-mm isotropic margin around the GTV to account for setup and patient uncertainties. The maximum dose of 33.3 Gy was used in the center, and a prescription dose of 18 Gy was applied to the 54% isodose around the PTV. Akinesia of the ocular globe was achieved by mechanical vacuum fixation, and pupil center deviation was continuously assessed during treatment via an infrared camera to stop dose delivery instantly in case of abnormal bulbus movement. Treatment was delivered in a single session. Follow-up after 6 and 12 months showed a reduction in the volume of the melanoma. No major complications were recorded.

This report details the case of one of the first patients to be successfully treated for uveal melanoma using the ZAP-X^®^ radiosurgery platform. The results demonstrate that this approach can be safely applied and shows potential indicating that it is an important alternative to existing treatment modalities.

## Introduction

Uveal melanoma is the most common primary intraocular tumor in adults with a mean age-adjusted incidence of 5.1 cases per million per year and primarily found in the Caucasian population [1]. It most commonly affects the choroid (90%), followed by the ciliary body (6%), and the iris (4%) [2]. Possible symptoms of the disease include photopsia, floaters, visual field loss, and/or visual deterioration [3]. Uveal melanomas present as a dome-shaped mass (75%) or have a mushroom configuration because of rupture of Bruch’s membrane (19%) and rarely present as a diffuse variant (6%). The lesion can be pigmented (55%), non-pigmented (15%), or have mixed color (30%), and be associated with retinal detachment (71%), intraocular hemorrhage (10%), or extraocular extension (3%) [1].

There are different treatment options, including transpupillary thermotherapy, photodynamic therapy, radiotherapy (plaque radiotherapy, proton beam radiotherapy, Gamma Knife® radiosurgery (Elekta, Stockholm, Sweden), CyberKnife® radiosurgery (Accuray, Madison, USA), and linear accelerator (LINAC)-based radiosurgery) and surgery (resection, enucleation, exenteration) [1]. Management of posterior uveal melanoma depends on location, extent, size, visual acuity at presentation, and health status [1]. Stereotactic radiosurgery (SRS) is one of the techniques used, the results based on prognosis are similar to those of other radiotherapy techniques, with a comparable survival rate to brachytherapy, proton beam irradiation, or enucleation [4-6].

The local tumor control, visual outcome, and survival of patients undergoing stereotactic photon beam radiation therapy are equivalent to those undergoing proton beam radiotherapy [7]. Regardless of the previously mentioned therapies, unfortunately, the 5-year mortality rate for uveal melanoma patients has not changed in the past 30 years [8]. Only the recently developed cancer immunotherapy, tebentafusp, which was approved in 2022, has the potential to extend the life expectancy of patients with metastasized uveal melanoma [9].

The ZAP-X^®^ system (Zap Surgical Systems Inc., San Carlos, USA) is a completely new type of dedicated self-contained and self-shielded surgical robot for SRS of the brain, head, and neck. SRS is a recognized procedure for the treatment of benign tumors like vestibular schwannoma, meningioma, pituitary adenoma, neuroma of other cranial nerves, and glomus-jugular tumors, and malignant tumors like brain metastasis, glioblastoma recurrence, and chondrosarcoma. It is also used for the treatment of trigeminal neuralgia and arteriovenous malformations. The ZAP-X^®^ Gyroscopic Radiosurgery System is designed to treat intracranial tumors of various sizes. Clinical studies have demonstrated its capability to effectively manage tumors as small as 0.04 cm^3^ [10].

The first ZAP-X^®^ platform was installed at Barrow Brain and Spine at St. Joseph's Hospital and Medical Center (Phoenix, Arizona, USA) and has been in clinical use since January 2019.

Due to the use of a 3.0 megavolt S-band LINAC and the integrated shielding, a radiation bunker is generally not required for the ZAP-X^®^. Akin to a large gyroscope, the LINAC is mounted within a combination of yoked gimbals with attached radiation shielding, each of which accurately rotates around a common isocenter. This mechanical construct enables the LINAC beam to potentially crossfire from approximately 2π steradians of solid angle, as is ideally required for cranial SRS.

Intrafractional patient motion is compensated by acquiring kV images around every 45 seconds, deriving the offset of the patient's head by comparison with digitally reconstructed radiographs and automatically moving the table. The Zap-X^®^'s image-to-image X-ray correlation technology enables precise spatial planning, and the combination of the system's technical features enables the precise delivery of ionizing radiation with a steep dose gradient [11]. In addition, the Zap-X^®^ has the first real-time megavoltage dose detector of its kind, which measures and verifies transit dosimetry [11,12]. The ZAP-X^®^ system works with a collimator wheel with eight circular apertures (ranging from 4 mm to 25 mm) and a source-axis distance of 45 cm to reduce radiation leakage and sharpen the beam penumbra [13].

SRS has become a well-established treatment for uveal melanoma. It has the advantage of a single-session delivery treatment without requiring invasive surgery for plaque implantation or prolonged hospitalization [14].

Herein we report one of the first cases of SRS of the eye using the ZAP-X^®^ platform in a patient with uveal melanoma, including a 12-month follow-up.

## Case presentation

A 78-year-old female patient visited the outpatient clinic of the Department of Ophthalmology, City Hospital Zurich, Switzerland. At the initial visit, the patient complained of a reduction in vision in her right eye for about three to six weeks. The visual acuity measurement revealed visual acuity of a hand movement in the right eye and a visual acuity of 20/25 in the left eye. Intraocular pressures were normal in both eyes. Anterior segment examination in each eye and fundus examination in the left eye were unremarkable. Her ophthalmic and past medical history was unremarkable. No allergies were reported.

Clinical examination

**Figure 1:**
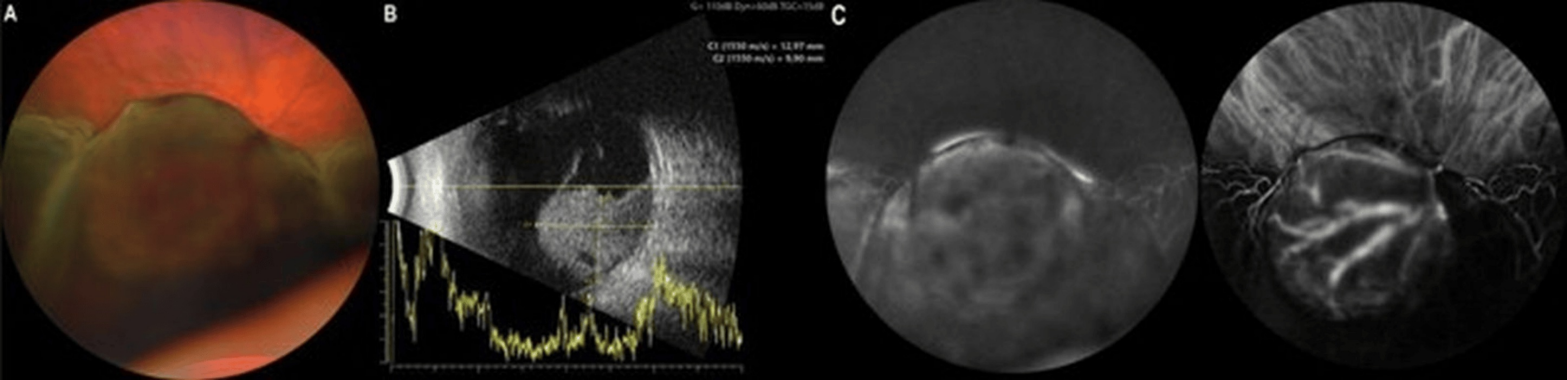
Multimodal imaging of the suspected choroidal lesion in the right eye at initial presentation. (A) Fundus: Color fundus photograph showing serous retinal detachment over the tumor (Zeiss Clarus 700, Zeiss Medical Technology, Jena, Germany). (B) B-scan ultrasonography showing a mushroom-shaped tumor with a maximum prominence of 12.97 mm (ABSolu Quantel Medical, Cournon-d'Auvergne, France) (C). Fluorescein and indocyanine green angiography (4:04 min, 102°) with suspected intrinsic vasculature (Spectralis, Heidelberg Engineering, Heidelberg, Germany).

Fundus evaluation of the right eye showed a serous retinal detachment in the inferior hemisphere without evidence of a retinal tear (Fig. 1A). Ultrasound (US) examination revealed a hyperechogenic mass with endophytic mushroom-shaped expansion into the vitreous cavity with a thickness of 12.97 mm and a largest basal diameter of 9.90 mm with associated neurosensory detachment. No evidence of an exogenous spread of the tumor was apparent ultrasonographically (Fig. 1B). Indocyanine green and fluorescein angiography could not confirm the suspected intrinsic vasculature with confidence due to interference from the overlying neurosensory retinal detachment (Fig. 1C).

In summary of all findings, a suspected diagnosis of uveal melanoma was made. The tumor was clinically classified using the eighth edition American Joint Committee on Cancer (AJCC 8th) melanoma staging system as T3a N0 M0. The patient was offered a stereotactic radiosurgery at the Swiss Neuroradiosurgery Center (SNRC). Staging workup to rule out metastases of uveal melanoma was done. No evidence of metastatic disease was found within the chest, intrapelvic, or intraabdominal areas on the whole‐body positron emission tomography/computed tomography (PET/CT) with 18F‐fluorodeoxyglucose (18FDG), and no metastases were seen on the magnetic resonance images (MRI) of the head. Complete blood count (CBC) and differential, comprehensive metabolic panel, and liver function test were all within normal ranges.

Pre-treatment imaging

**Figure 2:**
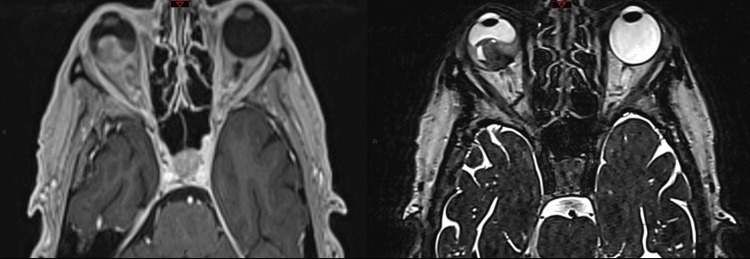
Magnetic Resonance Image - T1 (left), T2 (right). The pre-treatment magnetic resonance images demonstrate the endophytic intraocular mass without evidence of extraocular expansion.

First, a five-point thermoplastic mask (Klarity, Heath, USA) was constructed in order to establish a reproducible head position and immobilization. Akinesia of the ocular globe in the orbit was achieved by mechanical vacuum fixation after the application of topical anesthesia. A planning CT scan of the patient with the fixated ocular globe was acquired. Axial slice thickness 1.0 mm (CT Somatom, Top, FA. Siemens, Erlangen, Germany). For MRI, a gadolinium-contrast enhanced T1 of the orbit was acquired (1.0 mm multi-planar reconstruction (MPR)) and a T2 sequence with 0.5 mm slice thickness (3T-MRI Magnetom Vida, Siemens, Erlangen, Germany) (Fig. 2).

Treatment planning and delivery

**Figure 3:**
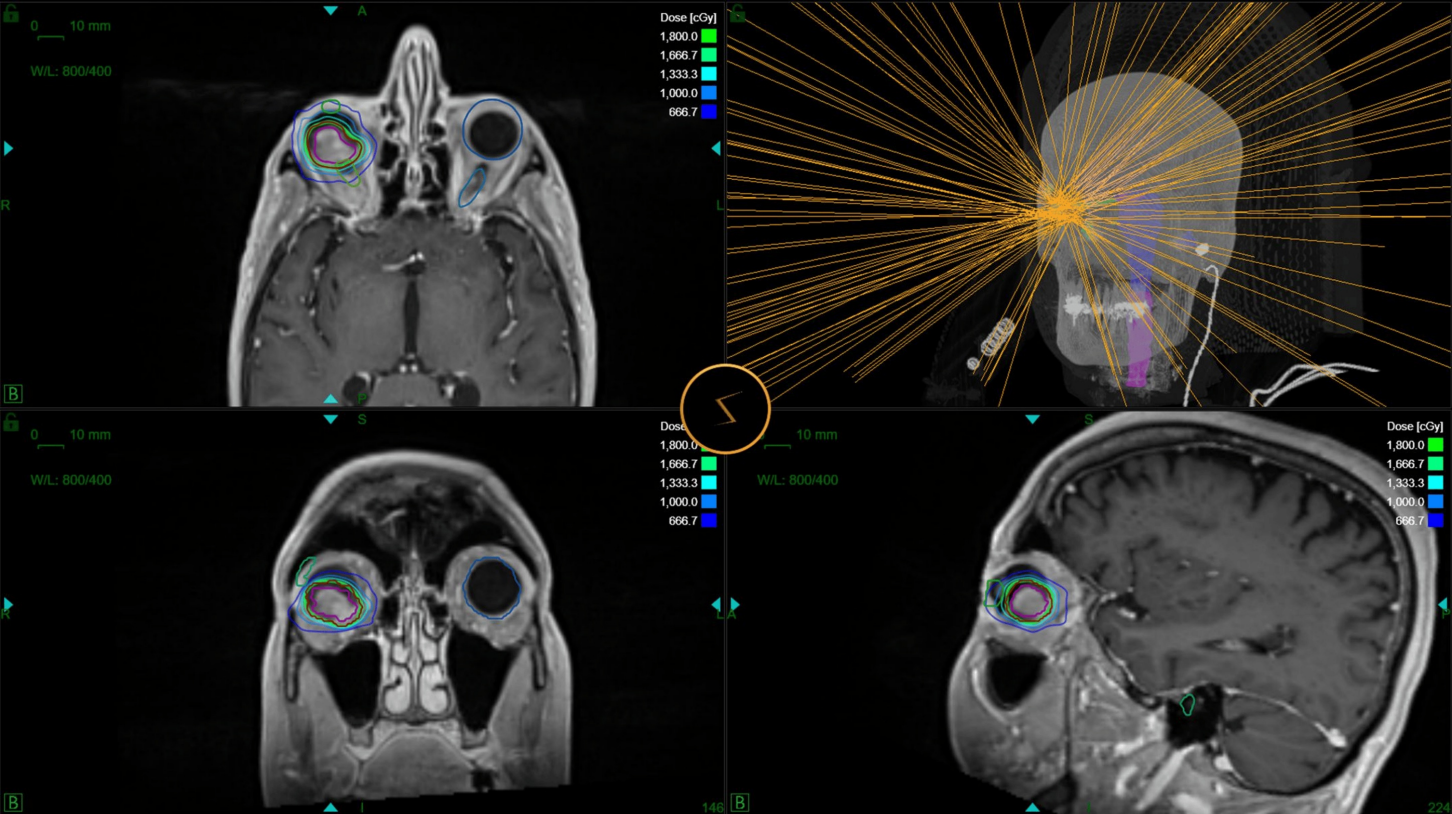
Zap-X® radiosurgery treatment plan. Isodose distribution around the PTV (red) in axial, coronal, and sagittal views. The green 54% isodose line represents the prescription dose of 18 Gy. PTV: planning target volume

MRI and CT datasets were imported into the treatment planning system (TPS), where the scans were overlaid to delineate the target volume (TV) and organs at risk. The GTV was defined by the ophthalmologist and radiation oncologist on T1 and T2 contrast-enhanced images, resulting in a volume of 1.47 cm^3^. To account for intrafraction motion and setup uncertainties, a 2-mm isotropic margin was added, resulting in a PTV of 3.34 cm^3^ (Fig. 3).

**Figure 4:**
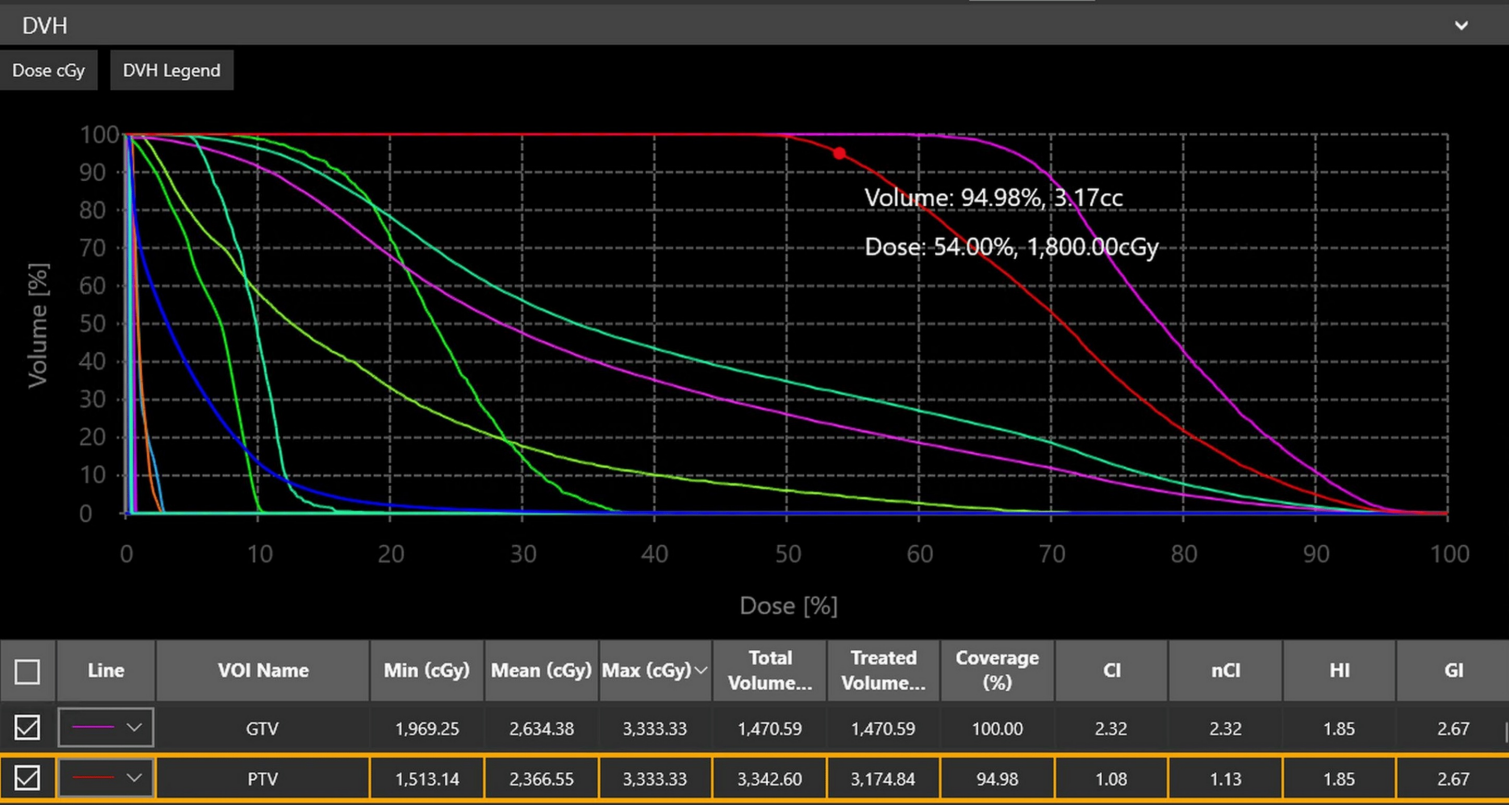
Dose Volume Histogram (DVH)

The treatment plan used eight isocenters (one with 15 mm, two with 10 mm, four with 7.5 mm, and one with 5 mm apertures) and 143 beams to cover 94.98% of the PTV of 3.34 cm3. The maximum dose of 33.3 Gy was used in the center and a prescription dose of 18 Gy was applied to the 54% isodose at the target margin. Treatment was delivered in a single session with the gyroscopic radiosurgery (GRS) device. The duration of the treatment was 37 min and 17 sec (Fig. 4).

**Figure 5:**
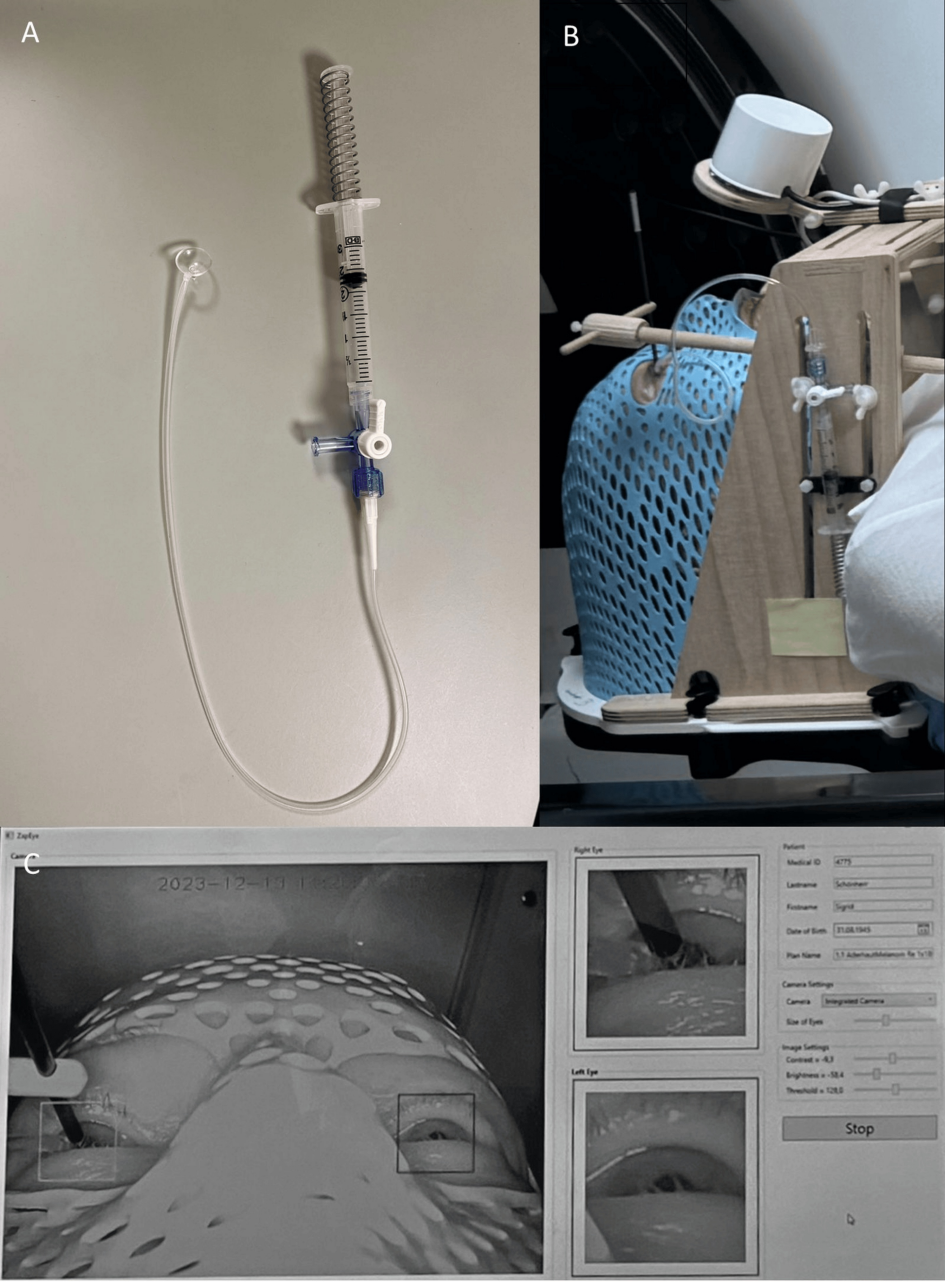
The non-invasive eye fixation device. (A) A suction cup connected to a 3 ml syringe to create a vacuum via a three-way stopcock. (B) The non-invasive eye fixation device consisted of a suction-coupled contact lens secured to the cornea and connected to a fixed frame and an infrared camera at the center. (C) The eye position was real-time monitored and the pupil was tracked with a camera. Figure credit: All images in this figure are owned by the authors.

After being informed in detail about the indication, the procedure, and the possible side effects of the treatment, the patient gave her verbal and written informed consent. Stereotactic radiation using the ZAP-X^®^ Radiosurgical Platform was performed on December 13th, 2023 at the Swiss Neuroradiosurgery Center (SNRC) in Zurich. Akinesia of the ocular globe in the orbit was achieved by mechanical vacuum fixation with a suction-coupled contact lens secured to the cornea and connected to a fixed frame. Tetracaine eye drops (tetracaine SDU Faure Gtt Opht 1%), which have the most substantial local anesthetic effect, were used [15]. No re-application was necessary over the course of treatment. Additionally, a potential pupil center deviation was recorded continuously during treatment via an infrared camera to stop dose delivery instantly in case of abnormal bulbus movement (Fig. 5) [16].

Post-radiosurgery examinations

**Figure 6:**
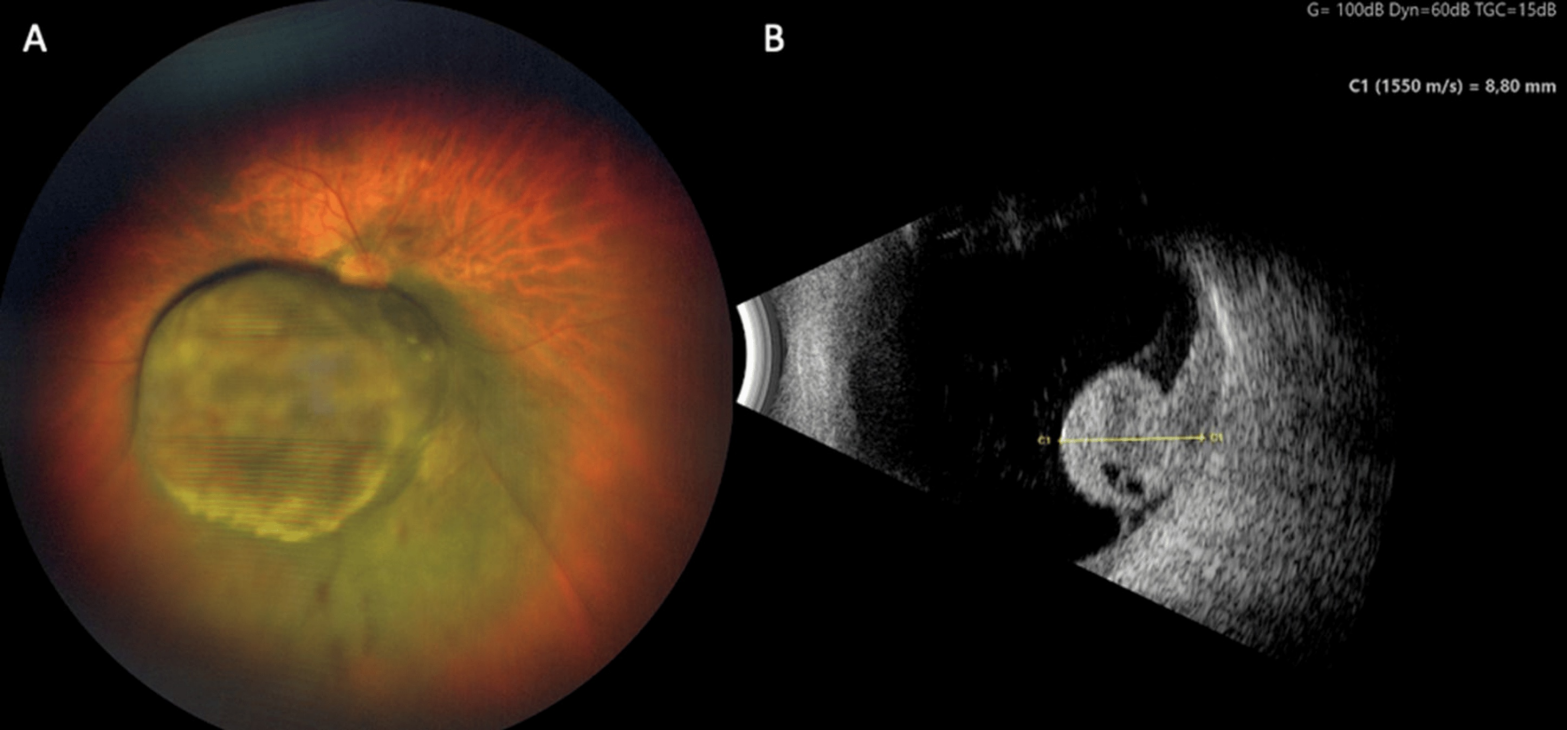
Images of the right eye 6 months after treatment. (A) Color fundus photograph (Zeiss Clarus 700, Zeiss Medical Technology, Jena, Germany) (B). B-scan ultrasonography showing a reduction of the prominence to 8.80 mm (ABSolu Quantel Medical, Cournon-d'Auvergne, France).

**Figure 7:**
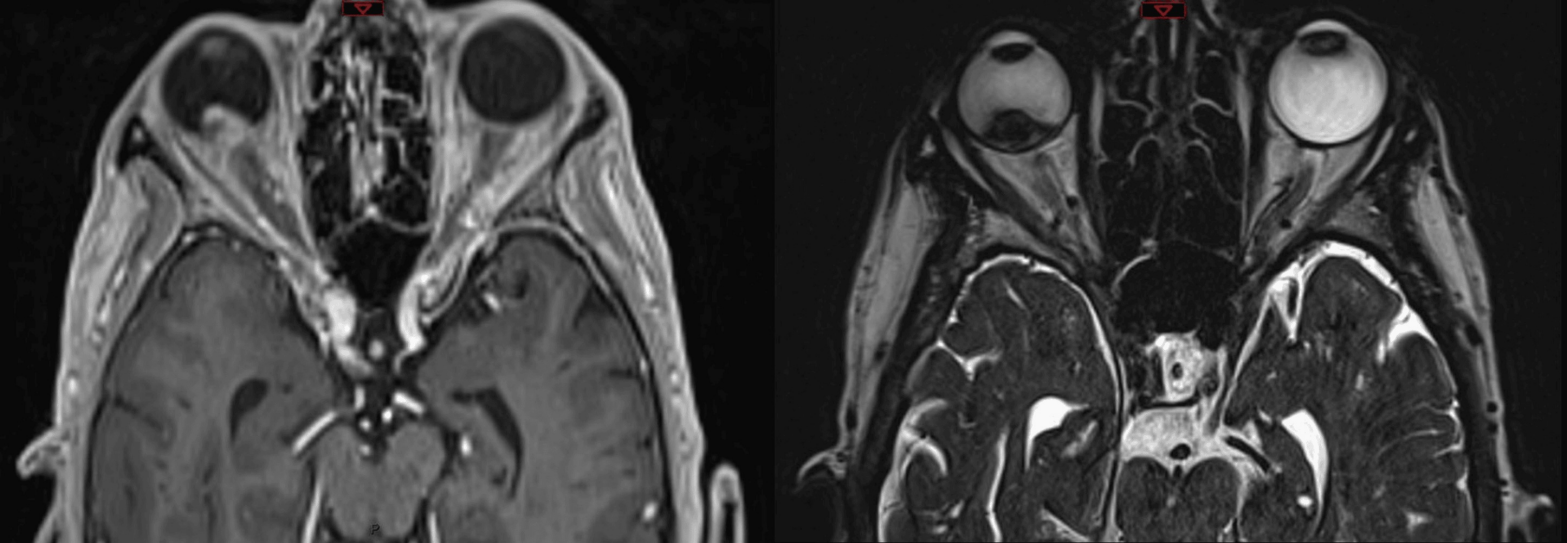
Magnetic Resonance Image - T1 (left), T2 (right). MRI images 6 months after treatment show a significant decrease in the size of the uveal melanoma in the right eye from the initial GTV of 1.47 cm^3^ to a GTV of 0.72 cm^3^, which is a reduction of 51%. GTV: gross tumor volume

There were no immediate post-radiosurgical complications and the patient was able to be discharged home after the treatment. The patient was treated with anti-vascular endothelial growth factor (VEGF)every 2 months, six times in total (Regeneron Pharmaceuticals, Inc, Tarrytown, USA, intravitreal ziv-aflibercept 1.25 mg/0.05 ml) and alternating with corticosteroids intravitreal every 2 months, five times in total (Sooft s.p.a., Fermo, Italy, Vitreal S 0.1 ml) to prevent neovascular glaucoma and to reduce possible radiation-induced ocular inflammation [17-19]. At a 5-month follow-up, visual acuity was hand movement and the intraocular pressure was 13 mmHg in the right eye. Anterior segment examination revealed a progression in the patient's cataract. Funduscopic examination revealed a still endophytic tumor in the vitreous cavity with still little serous neurosensory detachment of the juxtafoveal retina (Fig. 6A). B-scan ultrasonography showed a decrease in the tumor thickness from initially 12.97 mm to 8.80 mm after 6 months (Fig. 6B). MRI showed a significant decrease in the size of the uveal melanoma in the right eye from the initial GTV of 1.47 cm^3 ^to a GTV of 0.72 cm^3^, which is a reduction of 51% in comparison to the pre-radiosurgical imaging. There was no evidence of scleral invasion or extrascleral extension (Fig. 7).

**Figure 8:**
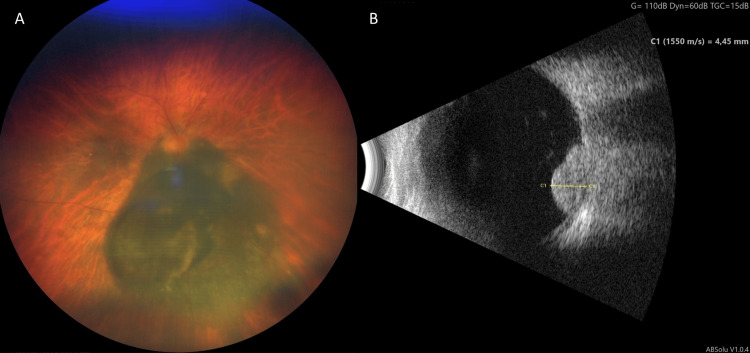
Images of the right eye 12 months after treatment. (A) Color Fundus photograph (Zeiss Clarus 700, Zeiss Medical Technology, Jena, Germany) (B). B-scan ultrasonography showing a reduction of the prominence to 4.45 mm (ABSolu Quantel Medical, Cournon-d'Auvergne, France)

**Figure 9:**
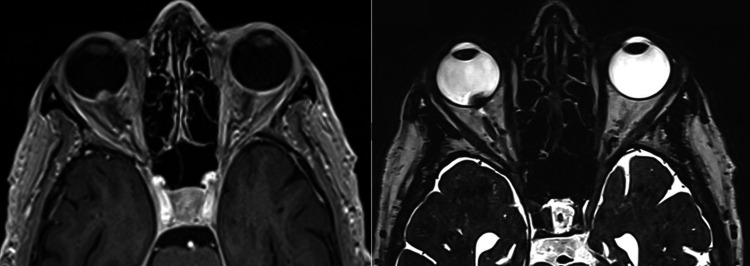
Magnetic Resonance Images - T1 (left), T2 (right). Twelve months after treatment, magnetic resonance images show a further decrease in the size of the uveal melanoma in the right eye from the initial GTV of 1.47 cm^3^ to a GTV of 0.24 cm^3^, which is a reduction of 84%. GTV: gross tumor volume

At a 12-month follow-up, the visual acuity was hand movement in the right eye with a significant cataract with a consecutive rise in her eye pressure to 38 mmHg without evidence of rubeosis iridis. The patient stated that she had no pain. B-scan ultrasonography showed a further decrease in the tumor thickness to 4.45 mm after 12 months (Fig. 8B). MRI showed a significant decrease in size of the uveal melanoma in the right eye from initial GTV of 1.47 cm^3^ to a GTV of 0.24 cm^3^, which is a reduction of 84% in comparison to the pre-radiosurgical imaging (Fig. 9).

## Discussion

The Collaborative Ocular Melanoma Study (COMS) has shown that there is no difference in 5-year all-cause mortality for large- and medium-sized choroidal melanomas in enucleation or brachytherapy [20]. Nowadays, it is standard practice to achieve local tumor control, minimize radiation-induced damage, and preserve the eye. In our case, the tumor was defined as large uveal melanoma depending on the COMS classification. Radiotherapy as a globe-sparing technique was the therapy of choice. Reported tumor control rates after SRS are 84-100%, with eye preservation in 78-97.4% of cases [21]. The justification for using the high-precision ZAP-X® delivery system to treat this patient was in order to offer the patient a therapy without the need for retrobulbar anesthesia and surgical intervention in an outpatient setting.

Proton beam therapy represents a well-established treatment option for patients diagnosed with uveal melanoma, particularly in Switzerland. The treatment procedure typically entails the placement of tantalum clips to ensure precise eye positioning during radiotherapy, followed by the administration of proton irradiation. The short-term burden associated with proton therapy in patients with uveal melanoma has rarely been addressed. In addition to an operation, the patient must spend several days to receive the fractionated proton therapy. The quality of life and treatment burden associated with this procedure differs significantly from that of ZAP-X^®^ SRS, which can be delivered in a single outpatient session [22]. In a quantification of dose perturbations induced by external and internal accessories in ocular proton therapy, Carnicer et al. could show that tantalum clips can produce important dose shadows (-20% behind the clip parallel to the beam and range reduction of 1.1 mm) and bands of overdose (15%) [23]. The much-cited advantage of the Bragg peak only comes into play in the direction of propagation - unfortunately, the dose distribution is not so sharply limited laterally. Ptaszkiewicz et al. described the dose perturbation behind tantalum clips in ocular proton therapy. The measurements of dose modification due to tantalum clips showed underdosing ranged from 4% to 32% [24].

In contrast to surgical enucleation of the ocular globe, SRS enables organ conservation without compromising overall survival. The utilization of SRS using ZAP-X^®^ obviates the necessity for retrobulbar anesthesia, thereby enabling the entire treatment to be conducted on an outpatient basis.

Regarding radiation therapy-related complications, only cataract formation was observed in our patient. The most common complications are cataracts, with a reported incidence ranging from 4% to 69%, and radiation retinopathy, reported in 5-68% of cases. The onset of radiation retinopathy occurs between 6 months and 3 years after radiation therapy [25]. Radiation-related complications are responsible for approximately half of secondary enucleations, the leading cause being neovascular glaucoma. A poor visual outcome is mainly associated with the presence of radiation retinopathy and radiation optic neuropathy [26]. There are currently no drugs approved for the treatment of radiation retinopathy. However, some drugs that have shown efficacy, although used off-label, include anti-VEGF agents and corticosteroids [27]. The therapy regime in our case to prevent radiation retinopathy and neovascular glaucoma is the use of anti-VEGF every 2 months (Regeneron Pharmaceuticals, Inc., Tarrytown, USA; intravitreal ziv-aflibercept 1.25 mg/0.05 ml) alternating with corticosteroids intravitreal every 2 months (Sooft s.p.a., Fermo, Italy; Vitreal S 0.1 ml). The 12-month results did not show any radiation retinopathy until now. The rise in eye pressure is due to the phacomorphic angel closure for which a surgery is already scheduled.

An essential requirement for accurate radiosurgery delivery is the immobilization of the ocular globe. In lieu of akinesia of the globe via retrobulbar anesthesia, we decided to utilize an eye stabilization device comprising a suction-coupled contact lens secured to the cornea and connected to a fixed frame. A potential eye deviation or suction loss was recorded continuously during treatment via an infrared camera to stop dose delivery instantly in case of abnormal bulbus movement. Thanks to the vacuum fixation, only subtle eye movement could be observed. The in-house-constructed eye stabilization device is simple to use and well tolerated by the patient. The possible risk of retrobulbar hemorrhage and globe penetration through retrobulbar anesthesia can be avoided [28,29]. Therefore, it is unnecessary to plan for an additional posterior margin to accommodate the posterior displacement of the eyeball resulting from the resorption of the 5 ml of anesthetic agent during the course of treatment.

It is challenging to identify an appropriate radiation dose that achieves effective local tumor control while minimizing the risk of radiation-associated complications. In the majority of cases, the literature indicates that a dose of between 18 Gy and 22 Gy is administered in a single session [30,31]. In our report, we employed a prescription dose of 18 Gy at the 54% isodose, which may be considered a potential limitation. Liegl et al. reported a local tumor control rate of 92.0% and 84.3% after three and five years, respectively, for a prescription dose of 21 to 22 Gy. When the prescription dose was 20 Gy or less, the local tumor control rate was 86.9% and 77.7%, respectively [14]. The 18 Gy dose at the 54% isodose resulted in an unusually high dose rate of 15 Gy/min, which has the potential to elicit a robust cellular reaction. Additionally, the mean dose GTV was 26.34 Gy.

For our PTV, a 2-mm isotropic margin was incorporated. In general, a margin of 2 to 3 mm is typically utilized for proton beam therapy, allowing for potential microscopic tumor extension, minor setup errors, eye movement during treatment, and beam penumbra [32]. The question of whether to include or exclude margins remains the most critical and controversial aspect of SRS. The review of Fiagbedzi et al. was conducted to examine the existing literature on the impact of margins in SRS for brain metastases. The findings indicated that an increase in margin addition is associated with an elevated risk of radio necrosis. Additionally, the local control rate demonstrated variability across different treatment modalities, underscoring the need for caution in making generalizations [33]. Foerster and colleagues used a 1-mm margin in their first case report about gyroscopic radiosurgery for uveal melanoma [34]. In light of the considerable dimensions of the uveal melanoma observed in our initial case, we elected to incorporate a 2-mm isotropic margin to address potential uncertainties.

The preliminary results presented here, based on a case report with a 12-month follow-up period, require further confirmation in a larger patient cohort. Nevertheless, the results are not unexpected, given that the Zap-X® has been developed to represent the most advanced radiosurgery for the head.

Owing to the limited sample size, a more robust understanding of the efficacy and safety of this treatment will require the analysis of additional cases. Further research with a larger cohort is essential to validate these preliminary observations. Nevertheless, this report on the treatment of a uveal melanoma with the ZAP-X^®^ stereotactic radiosurgery system underscores the system’s potential for the treatment of uveal melanoma. It is imperative to pursue a systematic approach to advancing the therapeutic potential of the ZAP-X^®^ SRS, aligning its applications with the specific indications under consideration.

## Conclusions

This case report demonstrates the successful use of the ZAP-X^®^ stereotactic radiotherapy system for the efficient and effective treatment of a uveal melanoma with a notable reduction in tumor size at the 6- and 12-month follow-up without any serious adverse effects. It is yet to be confirmed whether these results can be replicated in a larger patient cohort.
